# A Case of Nivolumab-Induced Multiorgan Toxicity: Concurrent Myocarditis and Interstitial Pneumonia

**DOI:** 10.7759/cureus.80732

**Published:** 2025-03-17

**Authors:** Shuntaro Fukushima, Eisuke Okamoto, Toshihiko Uchida, Tsunetaka Kijima

**Affiliations:** 1 General Medicine, Masuda Red Cross Hospital, Masuda, JPN; 2 Cardiology, Masuda Red Cross Hospital, Masuda, JPN; 3 General Medicine, Faculty of Medicine, Oda Training Center of General Practice, Oda Municipal Hospital, Shimane University, Oda, JPN

**Keywords:** immune checkpoint inhibitor (ici), immune-related adverse event (irae), interstitial pneumonia, myocarditis, nivolumab related adverse events

## Abstract

This is a rare case of nivolumab-induced multiorgan toxicity presenting as concurrent myocarditis and interstitial pneumonia in an 81-year-old patient with metastatic renal cell carcinoma. Immune checkpoint inhibitor (ICI)-associated myocarditis, a high-mortality immune-related adverse event (irAE), often presents with nonspecific symptoms, complicating early diagnosis, particularly when coexisting with other irAEs. In this case, the diagnosis was supported by elevated cardiac biomarkers, multimodal imaging findings (echocardiography, cardiac MRI, and coronary CT angiography), and electrocardiogram (ECG) abnormalities, including new-onset atrial fibrillation and right bundle branch block, indicative of myocardial involvement. High-dose methylprednisolone (1 g/day) was initiated on the second hospital day, followed by a gradual tapering regimen based on troponin trends and clinical improvement, leading to the resolution of myocarditis. This case underscores the importance of early recognition of ECG abnormalities as a diagnostic clue and highlights the diagnostic challenges of distinguishing myocarditis from other irAEs in patients receiving ICIs.

## Introduction

Immune checkpoint inhibitors (ICIs) are a class of immunotherapies that enhance the anti-tumor immune response by blocking inhibitory pathways in T-cell activation. Nivolumab, a programmed cell death protein 1 (PD-1) inhibitor, has been approved for the treatment of advanced renal cell carcinoma and is commonly employed as monotherapy or in combination with other targeted therapies [1]. While ICIs have revolutionized cancer therapy, adverse events associated with them stem from inappropriate activation of the immune response, which results in immune-related adverse events (irAEs) [2]. The incidence of single-agent irAEs ranges from 15% to 90% [3]. ICI-associated myocarditis is uncommon (0.27-1.14%) but has a very high mortality rate (up to 50%) [4]. On the other side, the frequency of ICI-associated pneumonia depends on the treatment regimen, with an incidence of 2.7-5% for all grades in patients treated with PD-1 inhibitors monotherapy, but mortality is rare (0.2%) [5]. irAE can affect any organ or organs [6], and the prognosis is poor when myocarditis, myositis, or neurotoxicity is present [7]. There have been few reports of complications with ICI-associated myocarditis and interstitial pneumonia [8,9]. We report a case of ICI myocarditis complicated by interstitial pneumonia that improved with early detection and treatment.

## Case presentation

An 81-year-old male with a history of renal cell carcinoma presented to our department in the evening. Eleven years prior, he underwent a right nephrectomy followed by surgery for lung metastases. He had refused chemotherapy but had no signs of recurrence. However, he was diagnosed with bone metastasis from renal cell carcinoma due to a pathological fracture of the femoral shaft three months prior. Consequently, nivolumab treatment was initiated one month previously (day 0) for bone metastasis. The patient received two doses at two-week intervals.

**Figure 1:**
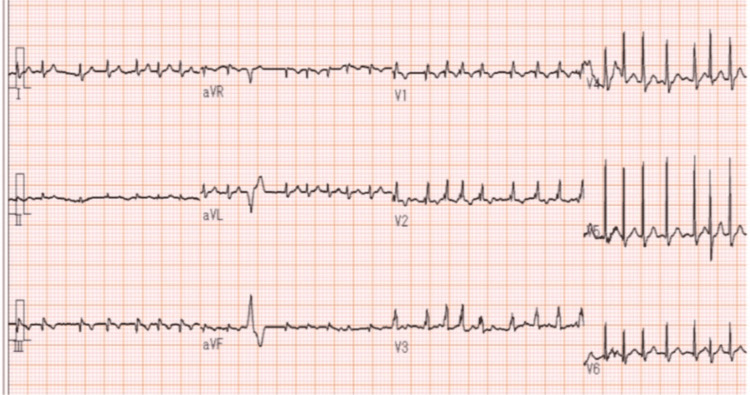
Electrocardiogram (ECG) findings Day 23 (day of admission): New-onset atrial fibrillation and incomplete right bundle branch block.

Seven days after the second dose (day 23), the patient presented for a routine visit with a fever and a persistent cough that had started several days earlier. A chest computed tomography (CT) scan suggested right lung pneumonia, and the patient was referred to our emergency department. He arrived ambulatory with no complaints of weight change, dyspnea, weakening of the muscles, or myalgia. He had a body temperature of 37.2°C, blood pressure of 122/85 mmHg, heart rate of 98 bpm, blood oxygen saturation of 95% on 1 L oxygen, and respiratory rate of 20 breaths per minute. We observed decreased breath sounds and fine crackles in the right lung, an irregular pulse, mild jugular venous distension, and bilateral lower-extremity edema. New-onset atrial fibrillation and incomplete right bundle branch block were noted on ECG (Figure 1).

**Figure 2:**
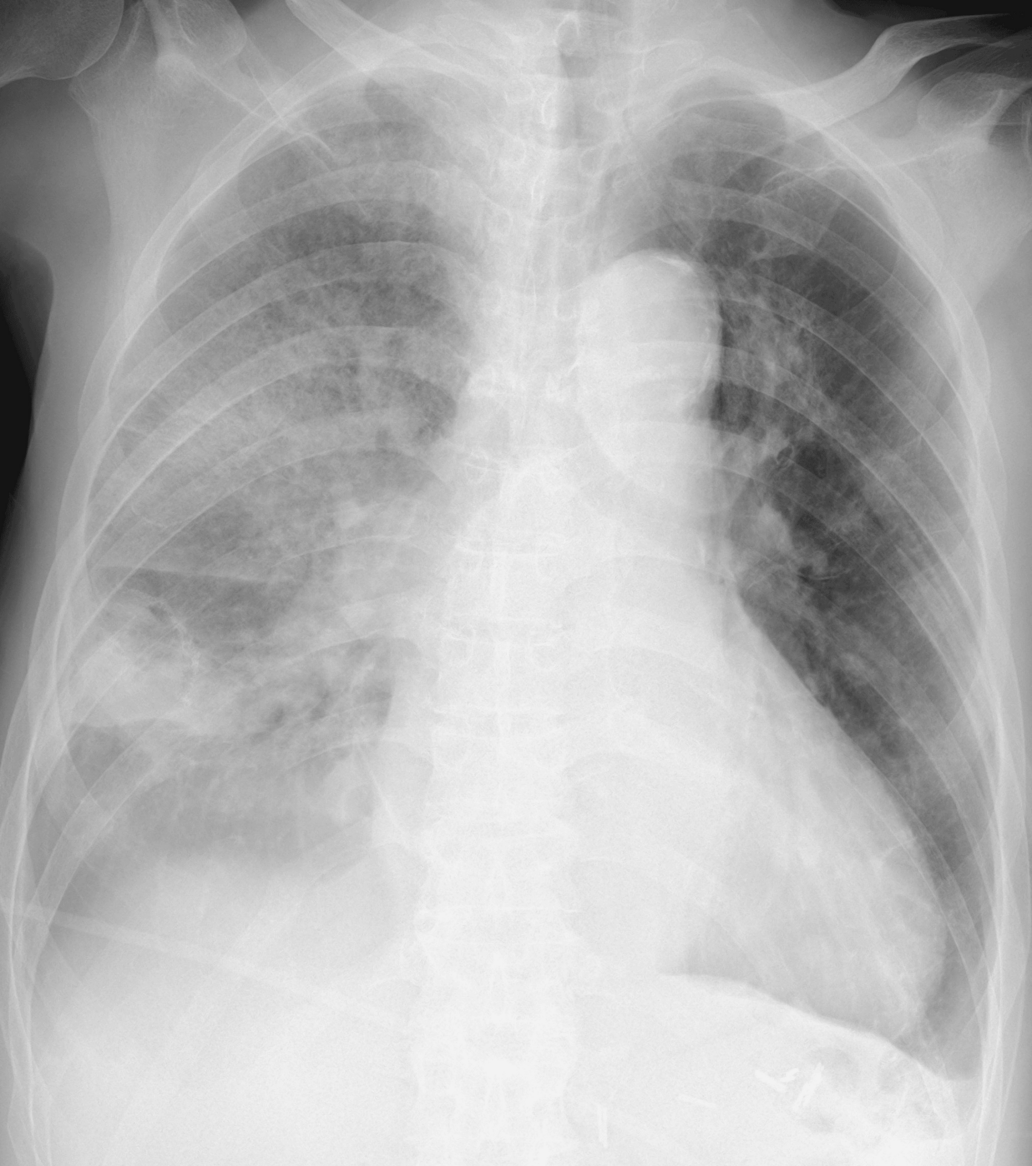
The chest X-ray The chest X-ray showed decreased transparency in the right lung and signs of pulmonary congestion.

Signs of pulmonary congestion and reduced transparency in the right lung were visible on the chest X-ray (Figure 2).

**Figure 3:**
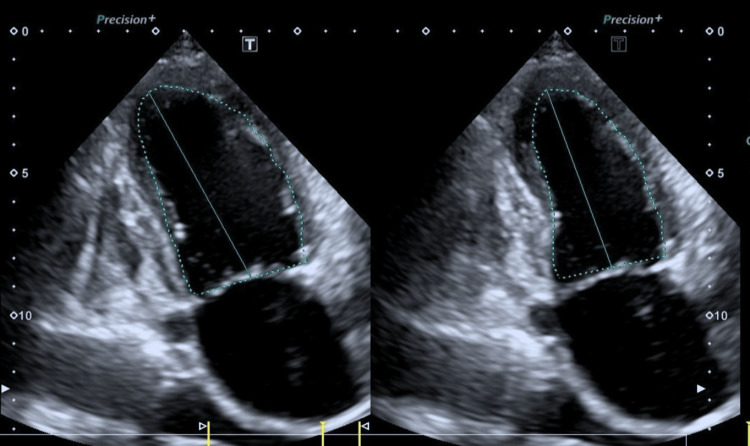
Echocardiography Diffuse left ventricular wall motion abnormalities (LVEF 46%), with a particular reduction in wall motion of the ventricular septum to the inferior wall, were seen in the echocardiography.

Echocardiography showed diffuse left ventricular wall motion abnormalities (LVEF 46%), with a particular reduction in wall motion of the ventricular septum to inferior wall, and mild pericardial effusion (Figure 3). 

Blood tests revealed an elevated high-sensitivity troponin I (hsTnI) level and increased inflammatory response (C-reactive protein), while creatine kinase (CK) levels were within the normal range (Table 1)

**Table 1:** Blood tests result

Investigation	Result	Reference Ranges
White blood cell count	7,000/μL	3,300-8,600/μL
C-reactive protein	11.24 mg/dL	<0.1 mg/dL
Lactate dehydrogenase	237 U/L	124-222 U/L
KL-6	306 U/mL	500 U/mL
B-type natriuretic peptide	457 pg/mL	<18.4 pg/mL
High-sensitivity troponin I (hsTnI)	982.4 pg/mL	<26.9 pg/mL
Creatine kinase	78 U/L	50-230 U/L
Thyroid stimulating hormone	6.62 μIU/mL	0.61-4.23 μIU/mL
Free thyroxine 4	1.04 ng/dL	0.88-1.5 ng/dL

**Figure 4:**
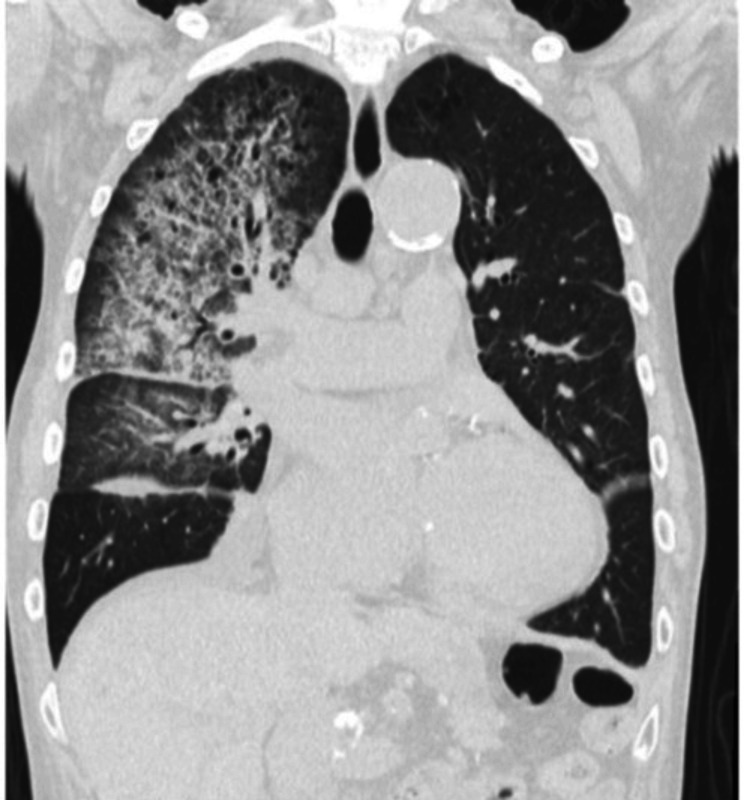
Chest computed tomography (CT) scan image CT scan shows ground glass opacities with bronchiectasis in the right upper and middle lung fields.

The polymerase chain reaction test for SARS-CoV-2 was negative. Chest CT revealed ground-glass opacities with bronchiectasis in the right upper and middle lung fields, indicating interstitial pneumonia (Figure 4).

**Figure 5:**
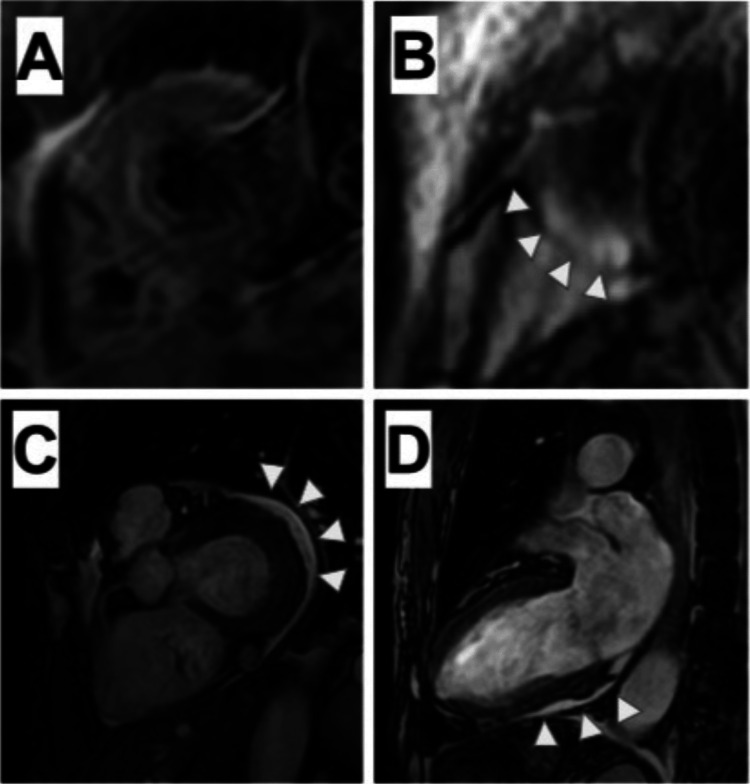
Cardiovascular magnetic resonance imaging (cMRI) A: dark blood T2-weighted cMRI does not show overt increased signal intensity; B: cMRI using a spin echo sequence 10 minutes after gadolinium injection shows mild late gadolinium enhancement (LGE) in the ventricular septum (arrowheads); C, D: cine MRI reveals pericardial effusion (arrowheads)

Coronary CT angiography revealed no significant stenosis in the coronary arteries or thrombus in the left atrial appendage, although mild coronary artery calcification was present. Cardiovascular magnetic resonance imaging (cMRI) did not show overt myocardial edema on T2-weighted images. However, mild late gadolinium enhancement (LGE) was observed in the ventricular septum. Additionally, cine MRI revealed pericardial effusion and left ventricular wall motion abnormalities (Figure 5).

These findings are suggestive of non-ischemic myocardial injury and support the diagnosis of myocarditis, meeting the supportive criteria of the updated Lake Louise criteria [10]. The clinical diagnostic criteria of the ESC 2022 guidelines were referenced [11]. In addition to troponin I elevation, the diagnosis was based on at least two minor criteria, including clinical syndrome (bilateral lower-extremity edema), a decline in left ventricular systolic function, and cMRI findings suggestive of myocarditis. The patient was diagnosed with nivolumab-induced myocarditis and interstitial pneumonia.

**Figure 6:**
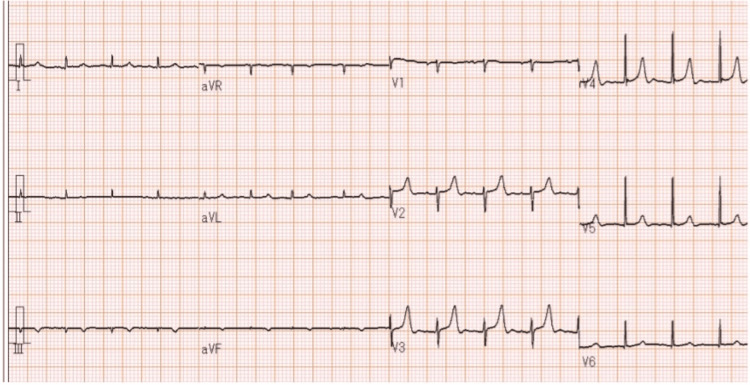
Electrocardiogram (ECG) findings Day 27 (fourth day in the hospital): After initiation of treatment, the ECG returns to sinus rhythm with a resolution of the right bundle branch block.

High-dose methylprednisolone (1 g/day) was initiated on the day after hospital admission. The apixaban was initiated as an anticoagulant for atrial fibrillation, with a CHA₂DS₂-VASc score of three, and the patient returned to sinus rhythm by electrical cardioversion. Since it was difficult to rule out bacterial pneumonia, antibiotics (tazobactam/piperacillin) were used in combination for seven days. Blood cultures were negative. The day after starting methylprednisolone, hsTnI levels improved to 270 pg/mL, and a chest X-ray showed improvement in pneumonia. Methylprednisolone was tapered to oral prednisolone 50 mg/day after three days. Follow-up echocardiography on the fourth day showed improved LVEF (56%) with recovery of wall motion in the ventricular septum, and ECG maintained sinus rhythm with resolved right bundle branch block (Figure 6).

**Figure 7:**
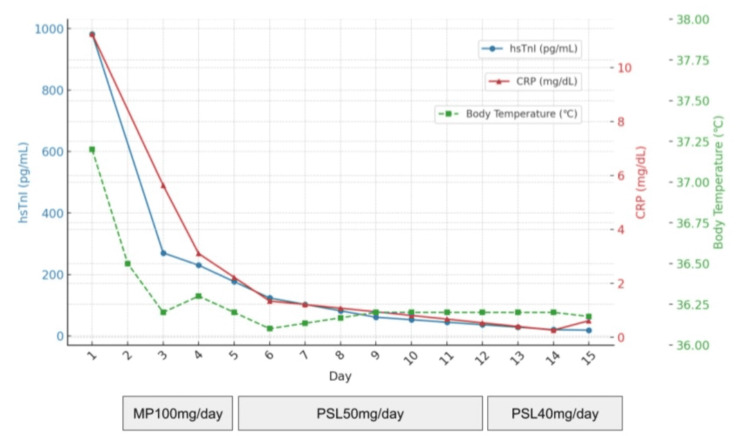
The clinical course of the patient since admission hsTnI: high-sensitivity troponin I; CRP: C-reactive protein; MP: methylprednisolone; PSL: prednisolone

The clinical course of the patient since admission is shown in Figure 7.

The patient’s condition improved steadily, and prednisolone was tapered. Prednisolone was reduced by 10 mg per week, and after the PSL 20 mg dosage, it was reduced at a rate of 5 mg per week and finally discontinued. No steroid-sparing agents were used during this course of treatment, and after approximately one year of follow-up, no recurrence of myocarditis, including electrocardiographic abnormalities or interstitial pneumonia, has been observed to date. The patient refused alternative chemotherapy, and the decision was made to provide palliative treatment if the carcinoma progressed.

## Discussion

This case underscores the need for vigilance in recognizing ICI-associated myocarditis, particularly in patients with seemingly non-cardiac presentations. The median time from the start of ICI monotherapy to symptom onset for myocarditis and pneumonia was 30 days (range from 18 to 60 days) and 2.8 months (range from nine days to 19.2 months), respectively [12,13]. This case also coincided with a predilection time of onset. 

The patient initially presented to the emergency department with pneumonia, but an early diagnosis of coexisting myocarditis was made based on abnormal ECG findings. ECG abnormalities are found in up to 89% of patients with ICI-associated myocarditis [4]. One multicenter registry reported a cumulative incidence of atrial fibrillation in 21.1%, atrial flutter in 1.4%, and multifocal atrial tachycardia in 2.1% of ICI-associated myocarditis patients [14]. Another study observed conduction abnormalities are highly prevalent, with 51% of patients exhibiting some degree of atrioventricular (AV) block, two-thirds of whom progressed to complete AV block [15]. Other conduction abnormalities, including sinus bradycardia, sinus arrest, and bundle branch block, have been reported [14,15]. If a new arrhythmia develops in a patient receiving ICI therapy, a diagnosis of myocarditis should be considered.

Elevated troponin is considered an important feature in almost all patients with ICI-associated myocarditis [4]. The higher the troponin level, the higher the risk of fatal outcome [16], but there is no clear cutoff to clarify the diagnosis. The degree of hsTnI elevation observed in this case was considered consistent with previously reported findings [17] in the early stages of myocarditis. In the ER setting, consider aggressively confirming troponin levels when ICI-treated patients have signs of heart failure, including ECG abnormalities. Importantly, when elevated troponin levels are detected, acute coronary syndromes should also be ruled out [4].

A combination of findings on cMRI, including the presence of one or more nonischemic (atypical) LGE, increased early enhancement, and focal or global increase in T2 signal intensity on cMRI (Lake Louise criteria) [10], has been used to support suspicion of myocarditis. However, cMRI has a high false-negative rate, and a myocardial biopsy should be considered when myocarditis is suspected, even if cMRI findings are negative [4].

ICI-associated myocarditis requires high levels of immunosuppressive therapy, such as high-dose corticosteroid pulse therapy or combination therapy with corticosteroids and other immunosuppressive agents. Thus, inadequate treatment may be given if myocarditis is not properly diagnosed, especially when other irAEs that can be treated with low-dose steroids are comorbid with myocarditis. Indeed, a report of fulminant myocarditis early after treatment of ICI-associated interstitial pneumonia indicates that insufficient doses of steroids may not suppress myocarditis [8].

Although there is no established treatment regimen or treatment duration, a retrospective study suggested that earlier and more intensive corticosteroid therapy is more effective in reducing myocardial damage [18]. Due to the long half-life of ICIs, long-term corticosteroid therapy and a gradual tapering strategy (typically over four to six weeks) should be considered [4]. In this case, the patient received treatment for eight weeks and has since maintained remission without further immunosuppressive therapy.

## Conclusions

ICI-associated myocarditis can coexist with various immune-related adverse events (irAEs), further complicating its diagnosis. Early recognition is crucial, and ECG abnormalities serve as an effective diagnostic clue, aiding in timely detection and management.
